# IMNI PRECISION trial protocol: a phase II, open-label, non-inferior randomized controlled trial of tailoring omission of internal mammary node irradiation for early-stage breast cancer

**DOI:** 10.1186/s12885-022-10454-1

**Published:** 2022-12-27

**Authors:** Wei-Xiang Qi, Lu Cao, Siyue Zheng, Cheng Xu, Rong Cai, Haoping Xu, Gang Cai, Jiayi Chen

**Affiliations:** Department of Radiation Oncology, Ruijin Hospital, Shanghai Jiaotong University School of Medicine, Shanghai, China

**Keywords:** Clinical trial, Clinical-genomic model, Breast cancer, Precision radiotherapy, Internal Mammary Node Irradiation

## Abstract

**Background:**

Since the publication of MA-20 and EORTC-22922 trials, chest wall (CW)/ whole breast (WB) irradiation + comprehensive regional nodal irradiation (RNI) with internal mammary node irradiation (IMNI) has been the standard adjuvant treatment for early-stage breast cancer (BC). However, one size does not fit all BC, and the risk of recurrence significantly varies among this patient population. In addition, whether all BC patients presented with one to three positive lymph nodes (pN1) could benefit from IMNI remains controversial. Thus, the optimal adjuvant RNI volume for early-stage BC with T1-2N1 remains undetermined.

**Methods:**

The IMNI PRECISION trial is a single institute, open-labeled, non-inferior, randomized controlled trial. A total of 214 clinically “high risk” BC patients which is characterized as having at least two of the five clinically adverse factors (age ≤ 40, three positive LN, T2 stage, grade 3 and Ki-67 index ≥ 14%), but genomic score “low risk” (the genomic score ≤ 44) N1 breast cancers are randomly assigned to omitting IMNI group (experimental group) or with IMNI (control group) with a 1:1 ratio. The primary endpoint of this trial is event-free survival, and secondary endpoints include overall survival and locoregional recurrence-free survival.

**Discussion:**

The IMNI PRECISION design allows promising clinical-genomic model to stratify the individualized risk of developing recurrence and guides the optimal RNI treatment for early-stage (pT1-2N1) BC patients. We anticipate that our results would provide high-level evidence to tailor IMNI according to individualized recurrence risk of BC.

**Trial registration:**

ClinicalTrials.gov Identifier NCT04517266. Date of registration: August 18, 2020. Status: Recruiting.

**Supplementary Information:**

The online version contains supplementary material available at 10.1186/s12885-022-10454-1.

## Background

In 2020, female breast cancer (BC) has become the commonly diagnosed tumor and the fifth leading cause of cancer-related death worldwide, with 2.3 million new cases and 685,00 deaths each year [1]. Two largest individual meta-analyses performed by Early Breast Cancer Trialists' Collaborative Group (EBCTCG) have found that adjuvant radiotherapy (RT) could significantly decrease the risk of developing recurrence and improve long-term outcomes of early stage BC patients underwent breast-conserving surgery(BCS) [2] or mastectomy [3]. Therefore, adjuvant RT plays an important role in the multidisciplinary management of early-stage BC. However, the optimal regional nodal irradiation (RNI) volume for early-stage BC remains controversial. In two large phase III randomized controlled trials (MA-20 and EORCT-22922), chest wall (CW)/whole breast (WB) + comprehensive RNI with IMNI significantly decreases the disease-free survival (DFS) and BC mortality for high risk N0 and pN1 BC patients [4–6]. Based on these results, comprehensive RNI with IMNI for pN1 patients is strongly recommended by the National Comprehensive Cancer Network (NCCN) guideline. On the other hand, in a recent phase III trial performed by Suh C.O. et al., the authors find that comprehensive RNI including IMNI does not significantly improve the DFS among node-positive BC patients, while subgroup analysis indicates that patients with primary tumor located with medially or centrally could might benefit from additional IMNI [7]. As a result, one size does not fit for all early-stage BC as this patient population is a heterogenous disease. Until now, the optimal RNI volume for pN1 BC remains undetermined.

Since IMNI would inevitably increase the lung and heart radiation dose, which might be related with an increased risk of developing cardiac or pulmonary toxicities [8]. Thus, in clinical practice, histological classification of breast tumors has been instrumental in determining a more personalized approach for pN1 breast cancer. Indeed, multiply studies have been performed to stratify clinical “high risk” early-stage BC patients who might be obtained from adjuvant post-mastectomy radiotherapy (PMRT) [9–14]. In consistent with those previous reports, our institution establishes and validates a prognostic nomogram including five risk factors (age, number of positive LN, tumor size, grade and Ki-67) accurately predict 5-year BC specific survival after mastectomy without adjuvant radiotherapy, which provides a practical stratification system to identify “high-risk” BC patients who might be indicated for adjuvant RT [15]. Subsequently, we validate this risk stratification model to individualize the adjuvant RT strategy among pT1-2N1 BC treated with modern systemic therapy, our result indicates that PMRT is an independent prognostic factor for DFS (HR 0.50, *p* = 0.05) among the clinical “high-risk” patients. However, those clinical “high-risk” patients could not benefit from comprehensive RNI with IMNI compared to those without IMNI (*p* > 0.05) [16]. In comparison with clinical risk model based on histopathologic factors, multi-genomic expression would provide individualized prognostic information for BC patients. Currently, multi-genomic expression profiling, such as 21-gene recurrence score and PAM-50-based 46-gene assay, has been recommended by international guidelines to tailor systemic therapy for patients with early-stage BC, but the clinical implications of multi-genomic expression for guiding radiotherapy is less well established. In a secondary analysis of a prospective trial of NSABP (National Surgical Adjuvant Breast and Bowel Project) B-14 and B-20,the authors find that the 21-gene Recurrence Score is an independent risk factor for locoregional recurrence in the cohort of 895 hormonal receptor (HR) positive patients treated with tamoxifen (HR2.16, 95%CI: 1.26–3.68, *p* = 0.005) [17]. In another retrospective analysis based on the Austrian Breast and Colorectal Cancer Study Group (ABCSG) 8 trial, the authors demonstrate that PAM-50 could be used to identify high local recurrence risk among postmenopausal HR-positive BC received with hormonal therapy, but it is not a predictive factor for obtaining from adjuvant RT [18]. RecurIndex, combined with 18 cancer genes and 10 reference genes, is a multigene profiling assay based on Chinese 28 genes, which could be used to predict the risk of local recurrence and distant metastasis for BC patients [19–22]. Our research team have demonstrated that the 5-year local–regional recurrence (LRR) free survival rates are 95–100% for N0 and N1 mastectomy BC patients with the 18-gene scores of < 44, while the LRR-free survival rate decreases to only 27–51% among BC patients with the 18-gene scores of more than 44 [23]. Thus, the 18-gene recurrence score might be used to identify biologically “high risk” breast cancer patients who would benefit from IMNI. Currently, to our best knowledge, there is no prospective studies to specifically investigate the role of IMNI in pN1 BC according to clinical-genomic model. As a result, we design this prospective trial by using an established clinical risk model combined with 28-gene score (RecurIndex) to individually stratify the risk of pN1 BC, and then to investigate whether those clinical “high-risk”, but genomic “low risk” pN1 BC patients would be safely omitting IMNI. Based on the Standard Protocol Items: Recommendations for Interventional Trials (SPIRIT) guidelines, our research team write the prospective trial protocol.

## Materials/design

### Hypothesis

We hypothesize that clinical risk model combined genomic model could individually define the risk of developing recurrence among early-stage BC patients, and those clinically high risk but genomic score “low risk” BC patients would be safely omitted from IMNI.

### Objectives

The primary objective of this prospective non-inferior, RCT aims to investigate whether clinically “high risk”, but genomic score “low risk” early-stage BC would be safely omitted from IMNI.

## Methods

### Trial design

**Fig. 1:**
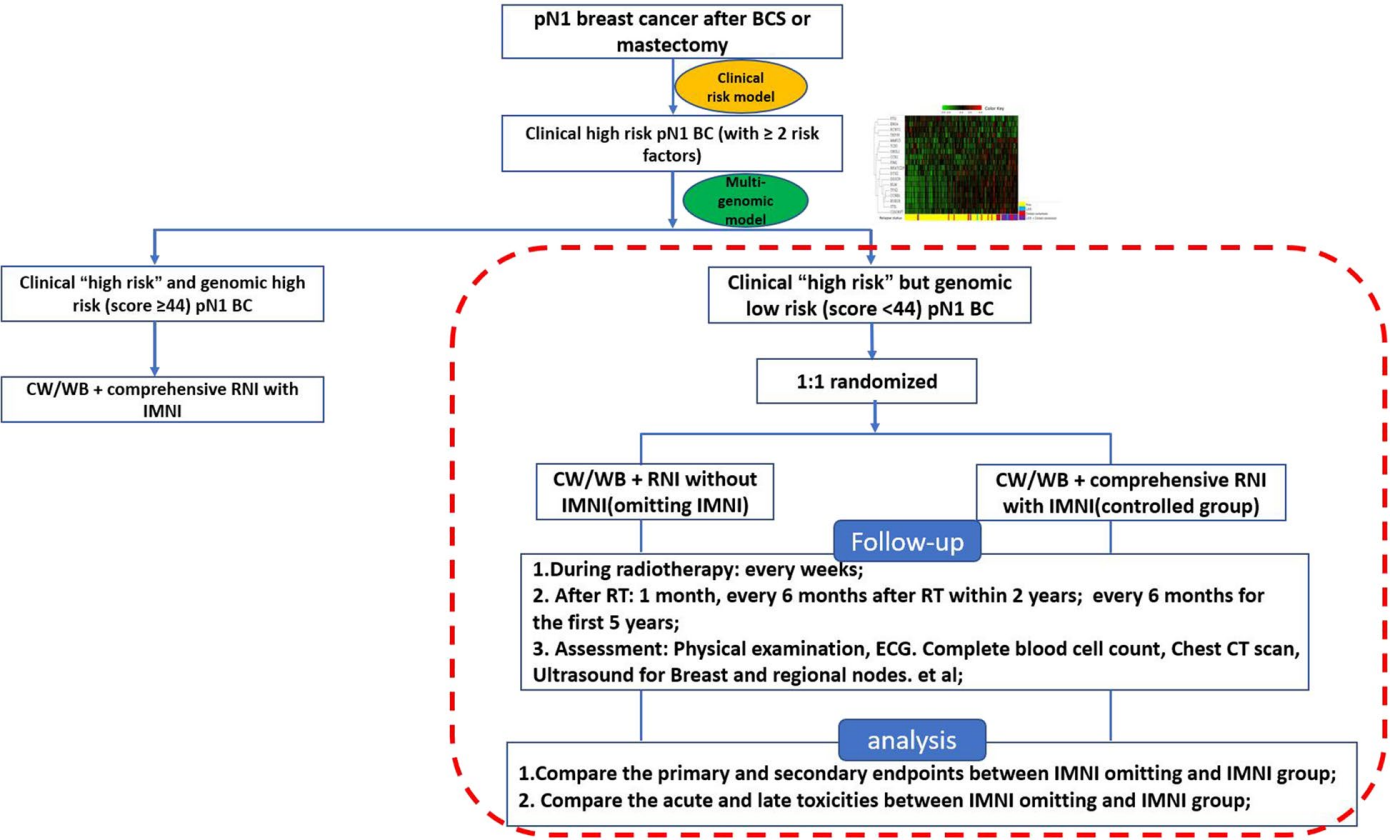
the design of the IMNI PRECISION trial

This is a prospective, non-inferiority, open-labeled, randomized controlled trial. We recruit histo-pathologically confirmed invasive breast cancer patient with pT1-2N1 after curative surgery from Ruijin Hospital, Shanghai Jiaotong university school of medicine. We initially use the clinical model to identify the clinically “high risk” of developing recurrence BC patients, defining as having at least two of the five clinical risk factors (age ≤ 40, three positive LN, T2 stage, grade 3 and Ki-67 index ≥ 14%). The formalin-fixed and paraffin-embedded (FFPE) tumor samples of those clinically “high-risk” BC would be detected using RecurIndex test, which is a multi-genomic test established according to Chinese 28 genes [19, 20]. Based on previous research, the accuracy of RecurIndex for predicting local–regional recurrence (LRR) in BC patients could be up to 93% by using 44 score as the threshold value for high or low risk. Therefore, patients with score ≤ 44 are classified as genomic low risk, > 44 are regarded as genomic high risk [23]. For early-stage BC patients with clinically “high risk” and genomic high risk, CW/WB + comprehensive RNI with IMNI would be recommended. For early-stage BC patients with clinically “high risk” but genomic low risk, these patients will be randomly assigned to RNI omitting IMNI (experimental group) or RNI with IMNI group (controlled group) with a 1:1 allocation ratio. Randomization was conducted with computer-generated random number by using SPSS software version 21.0 (IBM Corporation, Armonk, NY, USA). Participants will be followed until death or drop out of the study. Figure 1 shows the design of the IMNI PRECISION trial.

### Participants

To compared the non-inferior difference of 5-year event-free survival for RNI omitting IMNI vs. RNI with IMNI group among early-stage BC with early-stage BC patients with clinically “high risk” but genomic low risk, a total of 214 patients will be recruited.

The detailed instruction of present study would be offered to every potential participant before enrollment, and each enrolled patients should sign the informed consent.

### Inclusion criteria

(1) histologically diagnosed invasive carcinoma of the breast; (2) treated with radical surgery either mastectomy or BCS combined with axillary lymph node dissection or sentinel lymph node biopsy; (3) patients had 1–3 positive lymph node (pN1); (4) presented with at least 2 clinical risk factors; (5) female patients aged between 18 and 80 years old; (6) ECOG performance status ≤ 2 or Karnofsky ≥ 70%; (7) anticipated OS > 5 years; (8) Pathologically surgical margin is negative; (9)hormonal receptor, HER2 (human epidermal growth factor receptor 2) and Ki67 index can be tested for the primary breast tumor; (10) women of child-bearing potential should use adequate contraception for up to 1 month before enrolling the trial; (11) patients are able to understand and willing to participate the trial and signed consent form could be obtained.

### Exclusion criteria

(1) metastatic ipsilateral supraclavicular lymph node; (2) involvement of ipsilateral internal mammary lymph nodes; (3) Pregnant or lactating women; (4) patients received with breast reconstruction surgery; (5) Severe non-neoplastic medical comorbidities; (6) History of non-breast malignancy within 5 years with the exception of lobular carcinoma in situ, basal cell carcinoma of the skin, carcinoma in situ of skin and carcinoma in situ of the cervix; (7) simultaneous bilateral breast cancer; (8) had a history of radiotherapy to the neck, chest and/or ipsilateral axillary region; (9) presented with active collagen vascular disease; (10) presented with distant metastatic disease; (11) the primary breast tumor is unresectable; (12) Interval between radical surgery and radiotherapy was longer than 3 months or interval between last dose of adjuvant chemotherapy and radiotherapy was more than 8 weeks.

### Pre-treatment evaluation

1. History and physical examination: assessment of ECOG status, histology of cardiovascular disease, height, weight; comorbidities;
2. Blood test: blood cell count; liver and renal function; myocardial enzymes; thyroid function test;
3. Image examinations: breast ultrasonography or breast magnetic resonance imaging (MRI); chest CT, regional nodal ultrasonography; ultrasonography of liver;
4. Cardiac examination: Twelve-lead electrocardiogram; echocardiography;

### Radiotherapy

We contour the clinical target volume (CTV) on all CT slices if these structures are visible based on RUIJIN guideline [24], and contour the organs at risk (OAR) based on Radiation Therapy Oncology Group (RTOG) contouring guideline [25]. The recommended dose constraints for OARs and required dose coverage of plan treatment volume (PTV) are summarized in supplemental Tables 1 and 2.

### Interventions

#### Omitting IMNI group (experimental group)

Irradiation would be delivered to chest wall or whole breast combined with supra/infraclavicular nodes, and IMNI is omitting. Both hypofractionated or conventional intensity-modulated radiation therapy for BC are allowed. The hypofractionated prescribed dose is 40.05 Gy/15 Fx, with a tumor bed boost for breast conserving surgery (BCS) patients at a total dose of 10.68 Gy/4 fractions. And the conventional prescribed dose is 50 Gy/25Fx for CW/WB followed by a tumor-bed boost with 10 Gy/5Fx. It is recommended that 95% of the PTV covers 100% of the prescribed dose and the acceptable variation is that 90% of the PTV covers 100% of the prescribe dose, the maximum dose of the PTV should be less than 120% of the prescribed dose.

#### IMNI group (controlled group)

Irradiation would be delivered to chest wall or whole breast combined with comprehensive regional nodal areas including internal mammary node. The prescribed dose is the same as the omitting IMNI group, both hypofractionated or conventional intensity-modulated radiation therapy for BC are allowed. It is recommended that 95% of the PTV covers 100% of the prescribed dose and the acceptable variation is that 90% of the PTV covers 100% of the prescribe dose, the maximum dose of the PTV should be less than 120% of the prescribed dose.

### Outcomes

#### The primary outcomes

The primary outcome is the event-free survival, which is determined as the time from randomization to the time of a first recurrence in ipsilateral chest wall or in breast or in regional nodal or distant sites, a contralateral BC, or breast cancer specific death.

The secondary endpoints include the following outcomes: (1) local regional recurrence free survival, which is defined as the time from randomization to a first recurrence in ipsilateral chest wall or in breast or in regional nodal areas. (2) overall survival which is defined as the time from randomization to death from any cause.

#### Power and sample size calculation

The power and sample size are calculated according to the hypothesis that the 5-year event-free survival of omitting IMNI group is non-inferior to those patients treated with comprehensive RNI with IMNI. According to survival outcome of pN1 breast cancer in our institute, the 5-year event free survival (EFS) is estimated to be 91% for the control and 81% for experimental groups, respectively. The permissible limit for HR is 2.30, with statistical power of 80%, an alpha level of 0.025 (one-sided). With an anticipated enrolment period of 24 months, a total sample size of 208 patients is estimated to be needed. To compensate for eligible patients, the targeted number of patients is set at 214.

#### Allocation

Once obtaining a signed informed consent, each eligible participant would be randomized to omitting IMNI group or IMNI group (1:1) based on a computer-generated random number.

#### Treatment verification schedule

Daily alignment would be conducted by using skin markers for each participant. Online Cone-beam computed tomography (CBCT) was performed to verify the position with an action level of 5 mm for the first three fractions and weekly CBCT thereafter. In order to assure the quality of radiotherapy, the delineations and coverage requirement of CTV/PTV, and the dose constraints for OARs for the first 20 enrolled participants would be carefully checked by a senior radiation oncologist.

#### Data collection and management

By using an electronic data capture (EDC) system built by Ruijin hospital, all enrolled patients’ information would be collected and recorded, and only authorized and trained investigators would have the access to obtain these clinical data. If there is any amendments of the trial protocol, it should be reviewed and approved by the ethical committee of Ruijin hospital. Before the completion of the trial, auditing would be conducted every six months.

#### Follow up

After completion of radiotherapy, each enrolled patient would be followed up for at least 5 years. According to the follow-up program, the oncological outcomes and treatment-related complications will be assessed weekly during treatment and at each follow-up visit (1 month after RT, every 6 months after RT within 2 years; every 6 months for the first 5 years, and then annually) (Table 1). Any recurrence was cytologically or histologically confirmed whenever possible. According to the National Cancer Institute’s Common Terminology Criteria for Adverse Events version 3.0 (NCI-CTCAE 3.0) and the Radiation Therapy Oncology Group (RTOG) scale, acute and late radiation related toxicity during each follow-up visits would be recorded and graded.

**Table 1:** shows the follow-up schedule

	**STUDY PERIOD**									
	**Pre-radiotherapy**	**During Radiotherapy**	**Post-radiotherapy**							
**TIMEPOINT**	**0**	**Weekly**	**4w**	**6 m**	**12 m**	**18 m**	**2y**	**3y**	**4y**	**5y**
**ENROLLMENT:**										
Eligibility screening	X									
Informed consent	X									
Randomization	X									
**INTERVENTIONS:**										
Omitting IMNI		X								
With IMNI		X								
**ASSESSMENTS:**										
Physical Examination	X	X	X	X	X	X	X	X	X	X
Complete blood cell count	X	X	X	X	X	X	X	X	X	X
Thyroid function	X		X	X	X	X	X	X	X	X
Myocardial enzyme	X		X	X	X	X	X	X	X	X
Chest CT scan	X				X		X	X	X	X
Mammography					X		X	X	X	X
Ultrasound for Breast and regional nodes	X			X	X	X	X	X	X	X
ECG	X			X	X	X	X	X	X	X
Echocardiography	X			X	X	X	X	X	X	X

### Statistical analysis

Data will be analyzed in the “intention to treat” participants cohort. For the primary endpoint, Kaplan–Meier (K-M) analysis will be conducted to compare the EFS differences between omitting IMNI and with IMNI groups. Regarding the secondary outcomes of overall survival and locoregional recurrence free survival, K-M analysis and log-rank test would be performed. Descript analysis was used to summarize the baseline characteristics. Continuous variables were summarized by median and range, and qualitative variables were described as frequencies and proportion.

### Current trial status

The trial has been approved by the Ethics Committee of Ruijin hospital, Shanghai Jiao Tong University School of Medicine (RJ 2020–250) on 14 August 2020 and registered on ClinicalTrials.gov (NCT04517266) before recruitment. The currently used trial protocol version is Version 2.0 (December 2020). The recruitment of the trial is initiated on 10 December 2020. At present, the IMNI PRECISION trial remains at the recruiting stage and a total of 75 patients have been enrolled until 30 July 2022. We would publish the survival outcome of patients enrolled in the present trial after completing the trial and long-term follow-up.

## Discussion

Due to an increased survival benefit and decreased recurrence from adjuvant RT, adjuvant RT has become a standard treatment for early-stage BC patients. However, the application of adjuvant RT is related to an increased treatment-related morbidity and is a heavy burden for patients due to its costly and inconvenient. As a result, great efforts have been performed to minimize RT related toxicities in recent years, but most of those ongoing trials focusing on omitting adjuvant radiation in low-risk populations by using clinical-genomic model. However, whether clinical-genomic model could individually stratify the recurrence risk of pN1 breast cancer and tailor the RNI volume for those patients remains unknown. Prior to this trial, several secondary analyses of prospective trials have indicated that genomic scores, including 21-gene Recurrence Score [17, 26, 27] and PAM-50 genes [18], could be used as a prognostic tool to identify high local recurrence risk in node-negative or node positive HR-positive BC undergoing adjuvant hormonal therapy. Subsequently, prospective clinical trials, including PRIMETIME trial (NCT04916132), PRECISION trial (NCT05182437), EXPERT trial (NCT02889874) and IDEA trial (NCT02400190) et al., have been performed to investigate the prognostic role of multi-genes in guiding omitting RT among low-risk breast cancer after BCS. TAILOR RT trial (NCT 03,488,693) is the first large phase III trial to validate whether regional RT in biomarker low-risk node positive and T3N0 breast cancer could be safely omitted. However, no randomized controlled trial has been performed to guide RT in clinically high-risk BC patients presented with one to three lymph nodes positive. In this prospective study, we combine established clinical risk model with RecurIndex score to stratify the recurrence risk of pN1 BC, and classify them into two cohort. For those with clinical “high risk” and genomic “high risk”, CW/WB + comprehensive RNI with IMNI would be performed. While for those with clinical “high risk” but genomic “low risk”, patients would be randomized into omitting IMNI group or with IMNI group. In the era of precision medicine, we hope our research would provide high-level convincing evidence for IMNI for early-stage BC patients.

The current trial has several potential limitations. First of all, this is a single institute randomized controlled trial, and enrolling patients are performed at our institution, which might limit generalization of the result to other medical centers. Secondly, this is an open-label trial, both the participants and radiation oncologists are aware of the study group assignment, which might increase the selection bias. Thirdly, the primary outcome is 2-year event-free survival, and the follow-up is too short for observing events. After completing the trial, long-term follow-up of enrolled participants would be continued. Fourthly, the randomization of the present trial does not stratify according to risk factors, which might another source of heterogeneity. Finally, the 10% non-inferiority margin between the two groups might be larger in clinical practice, although this margin is discussed and decided by our clinical team.

In conclusion, the IMNI PRECISION trial is a non-inferior, randomized controlled trial to compare the survival and toxicity differences of omitting IMNI vs delivering IMNI in pN1 BC patients by using our established clinical risk model and multi-genomic model. We anticipate that our results would provide high-level evidence to tailor IMNI according to individualized recurrence risk of BC.

## Supplementary Information

**Additional file 1: Supplemental Table 1.** DVH constraints for PTV.

**Additional file 2: Supplemental Table 2.** DVH constraints for OARs.

## Data Availability

The individual de-identified participant data used and/or analyzed in the current study are available from the corresponding author on reasonable request.

## Abbreviations

CW: Chest wall
WB: Whole breast
IMNI: Internal mammary node irradiation
RNI: Regional nodal irradiation
BC: Breast cancer
EBCTCG: Early Breast Cancer Trialists' Collaborative Group
RT: Radiotherapy
NCCN: National Comprehensive Cancer Network
DFS: Disease-free survival
EFS: Event-free survival
OS: Overall survival
LRR: Local regional recurrence
PMRT: Post-mastectomy radiotherapy
NSABP: National Surgical Adjuvant Breast and Bowel Project
ABCSG: The Austrian Breast and Colorectal Cancer Study Group
FFPE: Formalin-fixed and paraffin-embedded
ALND: Axillary lymph node dissection
SLNB: Sentinel lymph node biopsy
ER: Estrogen-receptor
PR: Progesterone-receptor
HER2: Human epidermal growth factor receptor 2
BCS: Breast conserving surgery
EDC: Electronic data capture
CTCAE 3.0: Common Terminology Criteria for Adverse Events version 3.0 (CTCAE 3.0)
RTOG: The Radiation Therapy Oncology Group
