# Supplementary material for: IMNI PRECISION trial protocol: a phase II, open-label, non-inferior randomized controlled trial of tailoring omission of internal mammary node irradiation for early-stage breast cancer
Source: BMC Cancer. 2022 Dec 27;22:1356. doi: 10.1186/s12885-022-10454-1 (PMC9795778; doi:10.1186/s12885-022-10454-1)
Supplement: Supplementary file 1 — Additional file 1: Supplemental Table 1. DVH constraints for PTV. [file 12885_2022_10454_MOESM1_ESM.docx]

**Supplemental Table 1. DVH constraints for PTV**

| Structures | Constraints | Hypofractionated regimen | Conventional regimen |
| --- | --- | --- | --- |
| PTV of chest wall/breast + RNI | Per Protocol | D95%>40Gy | D95%>50Gy |
|  | Acceptable variation | D90%>40Gy | D90%>50Gy |
|  | Per Protocol | V43Gy<5% | V55Gy<5% |
|  | Acceptable variation | V45Gy<5% | V56Gy<5% |
|  | Per Protocol | V38Gy>99% | V48Gy>99% |
|  | Acceptable variation | V36Gy>99% | V45Gy>99% |
| PTV of breast +tumor bed boost+RNI | Per Protocol | D95%>50Gy | D95%>60Gy |
|  | Acceptable variation | D90%>50Gy | D90%>60Gy |
|  | Per Protocol | V55Gy<5% | V66Gy<5% |
|  | Acceptable variation | V58Gy<5% | V69Gy<5% |
|  | Per Protocol | V48Gy>99% | V57Gy>99% |
|  | Acceptable variation | V45Gy>99% | V54Gy>99% |

Abbreviations: DVH= dose volume histogram, PTV=planning target volume, RNI= regional nodal irradiation
