# Supplementary material for: IMNI PRECISION trial protocol: a phase II, open-label, non-inferior randomized controlled trial of tailoring omission of internal mammary node irradiation for early-stage breast cancer
Source: BMC Cancer. 2022 Dec 27;22:1356. doi: 10.1186/s12885-022-10454-1 (PMC9795778; doi:10.1186/s12885-022-10454-1)
Supplement: Supplementary file 2 — Additional file 2: Supplemental Table 2. DVH constraints for OARs. [file 12885_2022_10454_MOESM2_ESM.docx]

**Supplemental Table 2. DVH constraints for OARs**

| OARs | Hypofractionated regimen | | | Conventional regimen | | |
| --- | --- | --- | --- | --- | --- | --- |
|  | Dosimetric parameter | Per protocol | Acceptable variation | Dosimetric parameter | Per protocol | Acceptable variation |
| Heart for left-sided breast cancer | Mean | <5.5Gy | <6.5Gy | Mean | <7Gy | <8Gy |
|  | V25Gy | <10% | <15% | V30Gy | <10% | <15% |
|  | V8Gy | <20% | <25% | V10Gy | <20% | <25% |
| Heart for right-sided breast cancer | Mean | <2Gy | <3Gy | Mean | <2Gy | <3Gy |
|  | V4Gy | <15% | <20% | V4Gy | <15% | <20% |
| Ipsilateral Lung | Mean | <13Gy | <14Gy | Mean | <15Gy | <16Gy |
|  | V8Gy | <45% | <55% | V10Gy | <45% | <55% |
|  | V16Gy | <30% | <35% | V20Gy | <30% | <35% |
|  | V25Gy | <23% | <25% | V30Gy | <23% | <25% |
| Contralateral lung | Mean | <2Gy | <3Gy | Mean | <2Gy | <3Gy |
|  | V4Gy | <10% | <15% | V4Gy | <10% | <15% |
| Spinal cord | Max | <40Gy | N/A | Max | <45Gy | N/A |
| Ipsilateral Humeral head | Mean | <20Gy | <25Gy | Mean | <20Gy | <25Gy |

Abbreviations: DVH= dose volume histogram, PTV=planning target volume, RNI= regional nodal irradiation, CI=conformity index
